# Classification of User Emotional Experiences on B2C Websites Utilizing Infrared Thermal Imaging

**DOI:** 10.3390/s23187991

**Published:** 2023-09-20

**Authors:** Lanxin Li, Wenzhe Tang, Han Yang, Chengqi Xue

**Affiliations:** 1 School of Mechanical Engineering, Southeast University, 2 Southeast University Road, Nanjing 211189, China; lanxinliii7@gmail.com (L.L.); wenzhe.tang@seu.edu.cn (W.T.); 2 School of Instrument Science and Engineering, Southeast University, 2 Southeast University Road, Nanjing 211189, China; yh_wangyiyouxiang@163.com

**Keywords:** classification, emotional experiences, B2C website, infrared thermal image

## Abstract

The acquisition of physiological signals for analyzing emotional experiences has been intrusive, and potentially yields inaccurate results. This study employed infrared thermal images (IRTIs), a noninvasive technique, to classify user emotional experiences while interacting with business-to-consumer (B2C) websites. By manipulating the usability and aesthetics of B2C websites, the facial thermal images of 24 participants were captured as they engaged with the different websites. Machine learning techniques were leveraged to classify their emotional experiences, with participants’ self-assessments serving as the ground truth. The findings revealed significant fluctuations in emotional valence, while the participants’ arousal levels remained consistent, enabling the categorization of emotional experiences into positive and negative states. The support vector machine (SVM) model performed well in distinguishing between baseline and emotional experiences. Furthermore, this study identified key regions of interest (ROIs) and effective classification features in machine learning. These findings not only established a significant connection between user emotional experiences and IRTIs but also broadened the research perspective on the utility of IRTIs in the field of emotion analysis.

## 1. Introduction

In the digital era, the prominence of the e-commerce industry has been amplified remarkably, with business-to-consumer (B2C) websites being a crucial component. These platforms are designed to maximize benefits for both consumers and businesses [1]. A significant challenge for many businesses is to develop a B2C website that not only attracts a substantial customer base but evokes a positive emotional response [2]. Emotions significantly dictate an individual’s information processing, behavioral tendencies, and, ultimately, their decisions on final purchases [3]. Therefore, a well-designed B2C website that generates positive emotional experiences can effectively foster customer loyalty and recurring visits, thus enhancing the market competitiveness of the website [4]. To increase the purchasing rate for a given website, it is paramount to design a website that elicits a positive emotional response from users. This necessitates a comprehensive analysis of user emotional experiences, empowering designers with insights into customers’ buying behaviors and refining their website to become a strategic competitive asset.

**Figure 1:**
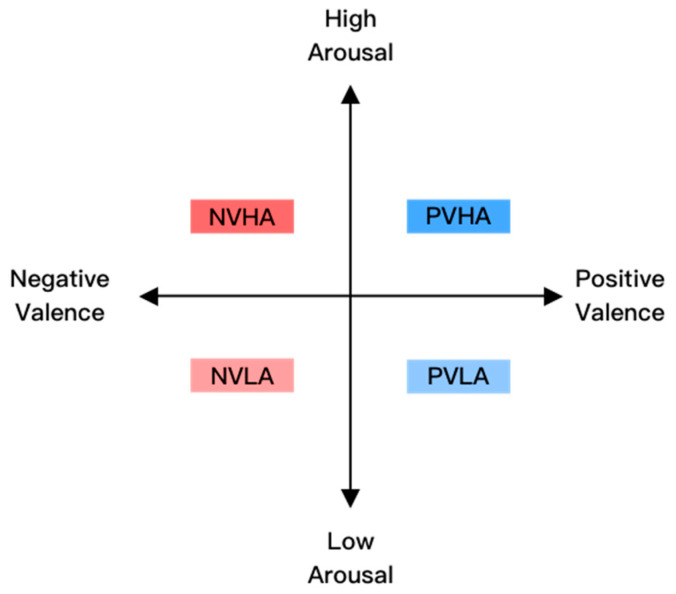
Two-dimensional emotion model.

Most researchers concur that emotions represent transient mental states influenced by life events [5,6,7]. Numerous models exist for quantifying emotion, with Russell’s circumplex model having gained significant traction in the field of human-computer interaction (HCI) [8,9,10]. This model conceptualizes an individual’s perception of their emotions within two-dimensional spaces, encompassing valence and arousal. These two dimensions are orthogonal and independent. Valence is positioned on the horizontal axis in this two-dimensional space. It is an individual’s judgment of whether an emotion is good or bad, representing the positivity and negativity of the emotion. Conversely, arousal, placed on the vertical axis, represents the level of emotional activation [11,12]. These two dimensions allow us to distinguish four basic categories of emotion, as shown in Figure 1. Other emotion models include Ekman’s discrete emotion model and Plutchik’s compound emotion model [13,14].

The measurement of emotional responses predominantly revolves around three approaches: subjective feelings, physiological reactions, and motor expressions [15]. In terms of expressive motion, numerous studies have been conducted on the recognition of facial expressions [16,17], utilizing a camera to capture visible light images of users during emotional reactions. Its performance is susceptible to variations in ambient lighting conditions [18]. Furthermore, a notable drawback of this approach is that individuals often tend to avoid changes in facial expressions when interacting with technological systems, resulting in reduced consistency between emotional experiences and facial expressions [19,20]. The Self-Assessment Manikin (SAM) is a subjective–affective report method based on the circumplex model that measures the degree of pleasure, arousal, and dominance of individuals in response to events through nonverbal pictorial assessment techniques, thus effectively mitigating the influence of individual differences in emotion cognition [21]. Many emotion research studies propose that the physiological changes in emotions are intimately linked with emotional experiences [22,23,24]. This theory has opened new avenues for analyzing emotional experiences on B2C websites. For instance, researchers have studied user emotional responses to two versions of a mobile phone interface using the physiological markers of electrodermal activity (EDA) and heart rate (HR) as indicators. The findings revealed higher EDA levels with the low-usability version, although the HR values showed no significant difference. Additionally, a correlation was observed between EDA, valence, and arousal [25]. Furthermore, additional methodologies include a multimodal approach combining eye movement indicators with the average galvanic skin response (GSR), skin temperature (SKT), and respiration rate (RSP) to assess user emotional experiences during online shopping. The findings indicated no significant differences in the GSR, SKT, and RSP responses, whereas the eye movement indicators showed significant variance [26]. In a separate investigation, researchers identified that distinct emotional states are associated with discernible variations in event-related potentials (ERPs), which means that users’ emotional experience while interacting with a website can be quantified by assessing the amplitude of the ERPs within relevant brain regions [27]. These studies highlight the diversity and effectiveness of methods that are currently employed to study emotional responses in the realm of B2C website interactions.

Physiological signals have shown promising results in detecting emotional experiences, as illustrated by the studies detailed above. However, these methods, while effective, require skin contact or are invasive. There is also the question of delay and cost, given the complexity of these procedures. In contrast, infrared thermal images (IRTIs) have recently gained recognition as a non-contact, non-invasive solution to evaluate human autonomic nervous activity and psychophysiological states. The autonomic nervous system (ANS) serves as the foundation for the thermal observation of emotion. It plays a pivotal role in regulating various physiological signals in individuals, and encompasses unconscious functions such as breathing, heart rate, perspiration, etc. Two biological mechanisms enable the thermal observation of emotions, namely subcutaneous vasoconstriction and emotional sweating, both of which can be characterized and quantified by IRTIs [28]. The advancement in IRTIs and the miniaturization of infrared detectors have incentivized numerous manufacturers to develop portable systems, specifically mobile and low-cost infrared thermal systems. This advancement has greatly facilitated experimental research [19].

In recent years, IRTIs have been widely used in the field of emotion recognition. For instance, IRTIs have been used to study changes in nasal temperature induced by feelings of guilt in children [29]. In the dimensions of valence and arousal, thermal images were used to mark physical changes during emotional tasks, revealing a link between nose temperature and emotions, particularly valence. Positive valence and arousal patterns led to an increase in nose temperature, while negative valence triggered a decrease [30]. Machine learning has also been incorporated into thermographic emotional studies, demonstrating high accuracy. For example, using the Stroop test to provoke stress, researchers recorded thermal imaging, cardiac, electrodermal, and respiratory activity. A support vector machine (SVM) model was employed for classification, and it was found that stress identification through IRTIs alone achieved a success rate of 86.84% [31]. Furthermore, the gray-level cooccurrence matrix (GLCM) features of thermal images have been explored for their potential use in emotion detection [32]. Therefore, the thermal imaging method combined with classification models could provide potential improvements in the quality and efficiency of website emotion evaluation. However, it is worth noting that previous studies predominantly employed videos and images as experimental stimuli. We tried to apply emotion classification based on IRTIs to the field of HCI and used B2C websites as the experimental stimuli.

This paper aims to investigate the effectiveness of the noninvasive IRTI method in classifying user emotional experiences when using B2C websites. We prepared an experimental setup wherein the emotional experiences of users were induced by websites with adjusted usability and aesthetic elements. The participants completed corresponding tasks and SAM, which provided the base truth of their emotional experiences and later served as labels in machine learning. This study is principally focused on establishing the potential of IRTIs in the context of HCI, particularly in its application for B2C websites.

The insights gained in the study will contribute to the understanding of user experience evaluation metrics, which are increasingly being employed as performance indicators for B2C websites. Additionally, they will facilitate the modeling of user emotional experiences from an HCI perspective. More pragmatically, this study serves to further comprehend the impact of website design elements on the emotional experiences of users, thereby enabling designers to optimize these elements for better user engagement. In achieving this, a cross-subject classification model that is promising for improved generalizability was developed. This model aimed to predict the emotional experiences of all participants rather than simply training a different model for each individual.

## 2. Methods

### 2.1. Design

This experiment was designed to demonstrate that IRTIs could be used to classify emotional experiences in HCI. This experiment adopted a 2 × 2 two-factor within-subject experimental design, with two independent variables: interface usability (high or low) and interface aesthetics (high or low). The dependent variables were emotional experience (valence and arousal) and the participant’s facial thermal responses. The emotional experiences were classified into positive emotional experiences and negative emotional experiences. As well, we also measured baseline (without emotional stimuli) thermal images for an actual comparison with the experimental conditions [28].

### 2.2. Participants

This experiment was conducted with a group of 24 students (12 males and 12 females) from Southeast University, and the age range was 19–25 (M = 22.50 and SD = 2.02) years old. The participants who accepted the experimental conditions were informed of the start time of the experiment 5 days in advance. Based on the study guidelines, the participants were required to abide by the following rules: no drinking alcohol 24 h before, no drinking coffee or smoking 3 h before, no application of lotions, cosmetics, antiperspirants, or shaving cream on the day of the experiment, and no facial obstructions such as hair and glasses. The participants were informed of the purpose and process of the whole experiment, and those who accepted the guidelines signed a letter of informed consent [33]. All of the experimental procedures of this study were approved by the clinical research Independent Ethics Committee of Zhongda Hospital affiliated with Southeast University (2022ZDSYLL128-P01).

### 2.3. Apparatus

The experiment was performed in a 5 × 5 m area in the Ergonomics Laboratory of Southeast University. To maintain a constant temperature, an air conditioner was used to keep the room temperature at 22 ± 2 °C and the relative humidity from 50 to 60%. In addition, the room was not directly ventilated or exposed to direct sunlight. The schedule was arranged between 9 a.m. and 3 p.m.

A FLIR ONE Pro (TeledyneFLIR LLC, Wilsonville, OR, USA) was used to obtain the thermograms; this device has a thermal sensitivity of 70 mk, a thermal pixel size of 12 µm, an infrared resolution of 160 × 120 pixels, and a spectral range between 8 and 13 µm. A One Fit^TM^ connector, which can flexibly connect to a phone to directly display thermal images on the screen, was implemented. The acquired thermal images were grayscale images with pixel intensities ranging from 0 to 255. Thus, higher temperatures were associated with brighter pixels (white areas indicated the hottest areas), and lower temperatures were associated with darker pixels (black areas indicated the coldest areas). The time interval for capturing a single frame of a thermographic image was 4 s. A FLIR ONE Pro was connected to a phone, and a tripod was used to secure the camera and phone at a distance of 1 m from the participant under study. Stimuli display and thermogram processing were performed using a MacBook Pro (13-inch, 2017, two Thunderbolt 3 ports) with a 2.3 GHz dual-core Intel Core i5 processor.

### 2.4. Stimuli

Usability and aesthetics were manipulated to evoke emotional experiences [34]. For usability, information architectures (IAs) primarily concern the organization and simplification of information, as well as the design and construction of information spaces. They were proposed to assist individuals in gaining a better grasp of information and making optimal decisions [35]. The relationship between the quality of IAs and usability has been well-researched [36]. Therefore, this experiment constructed two different IAs to manipulate usability. Afterward, the established IAs were compared based on the applications of latent semantic analysis (LSA) provided by the University of Colorado at Boulder [37]. We calculated the information scent of each navigation path to target or non-target. The LSA results are shown in Table 1, which illustrates that, in instances of good IAs, there is a high information scent associated with navigating toward targets and a low scent when navigating toward non-targets. Conversely, in cases of poor IAs, the information scent is a low scent for both target and non-target navigation. This results in the generation of high-usability websites and low-usability websites.

**Figure 2:**
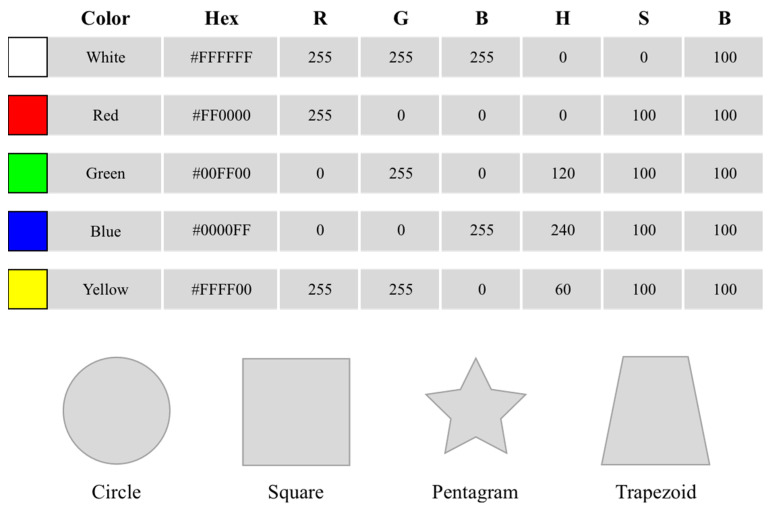
Background colors and product display shapes for the websites.

**Figure 3:**
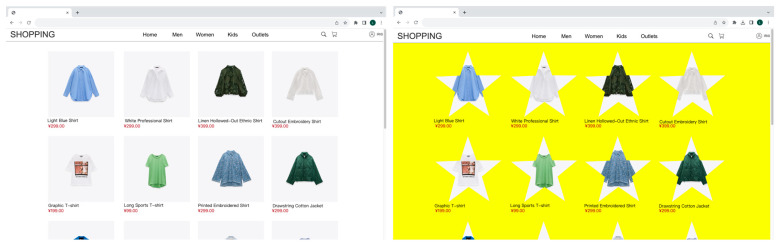
The high aesthetic (**left**) and low aesthetic (**right**) websites.

For aesthetics, 4 professors with over a decade of cumulative experience in website design were invited to engage in website design. They were tasked with choosing the simplest and most popular template among 10 websites. Then, adjustments were made based on this template. Afterward, according to research [38] and as shown in Figure 2, 5 kinds of website background colors and 4 kinds of product display shapes were combined to generate 20 websites. In a preliminary online study, 148 users were invited to rate the attractiveness of the websites using a 9-point scale. A total of 146 valid questionnaires were collected. We paired those with the highest and the lowest mean aesthetic scores, t (145) = 14.86, *p* < 0.01, Cohen’s d = 2.15. The yellow pentagram combination was selected as the website with the lowest aesthetic value (mean aesthetic score = 3.55, SD = 1.89), and the white square combination was selected as the website with the highest aesthetic value (mean aesthetic score = 7.35, SD = 1.63), as shown in Figure 3.

To evaluate the emotional experiences of users, an online shopping platform was implemented. There were 5 buttons on the navigation bar at the top of the website. Clicking on the “home” button returned the user to the first page, while the other buttons could be clicked to allow the user to select products through a drop-down navigation bar. The first-level navigation bar on the first page (except the “home” button) could be clicked to display a second-level navigation bar with 4 categories. Each of these categories could be clicked to display a third-level navigation bar on the right side, also displaying 4 categories. Clicking on any of these categories displayed a product list of the current category arranged in a 4 × 5 matrix. The pictures, names, and prices of the products were displayed in turn. Clicking on the product picture would display more details of the product. This online shopping website had a total of 1280 products.

Based on the obtained high-usability and low-usability navigation and high and low aesthetics scores, four websites were generated: high usability and high aesthetics (U+A+), high usability and low aesthetics (U+A−), low usability and high aesthetics (U−A+), and low usability and low aesthetics (U−A−). The websites only differed in the IAs of the navigation bar, the background color, and the product display shape; other elements were not changed.

### 2.5. Procedure

**Figure 4:**
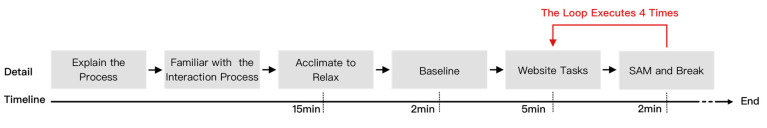
The procedure of the experiment.

The overall procedure of the experiment is shown in Figure 4. A participant was invited into the room upon arrival and was seated in a comfortable seat. The height of the seat was adjusted to ensure that the face of the participant was centered on the phone screen without movement; a distance of 1 m between the participant and the phone was verified.

Before starting the study, the participant received an explanation of the whole experimental process and the meaning of the SAM, which is scored on a scale from 1 to 5 and consists of the valence (positive and negative) and arousal (intensity) dimensions. The SAM result was considered the ground truth of the emotional experiences.

A template was provided to the participants before the formal experiment in the same way as the experimental stimulus interaction but without any experimental elements. Thus, the participants were able to familiarize themselves with the utilization of the website. Afterward, the participants were allowed to relax for 15 min to adapt to the environment and stabilize their body temperature. The baseline thermal responses of the participants were measured for 2 min. The baseline served as the foundation for defining the directionality of physiological changes during the emotional arousal process [28]. During the baseline measurement process, participants were instructed to rest and empty their minds of all thoughts, feelings, and memories [39].

After completing all of the steps described above, the participants completed 4 tasks on the B2C websites. For displayed product pictures, participants were asked to find the corresponding products and add them to the shopping cart. The participants searched for different products on the 4 websites to avoid operational memory interference. To eliminate effects related to the order in which the websites were presented, the stimulus order in this experiment was counterbalanced using a Latin square design [40], and the participants were asked to complete the corresponding task on each website in 5 min. If a participant did not complete the task in 5 min, they were asked to stop immediately. After completing all of the tasks for a particular website, the participants were required to complete the SAM, followed by a 2 min break to allow their state to return to baseline.

### 2.6. Thermal Data Processing

#### 2.6.1. Infrared Thermal Image Preprocessing

**Figure 5:**
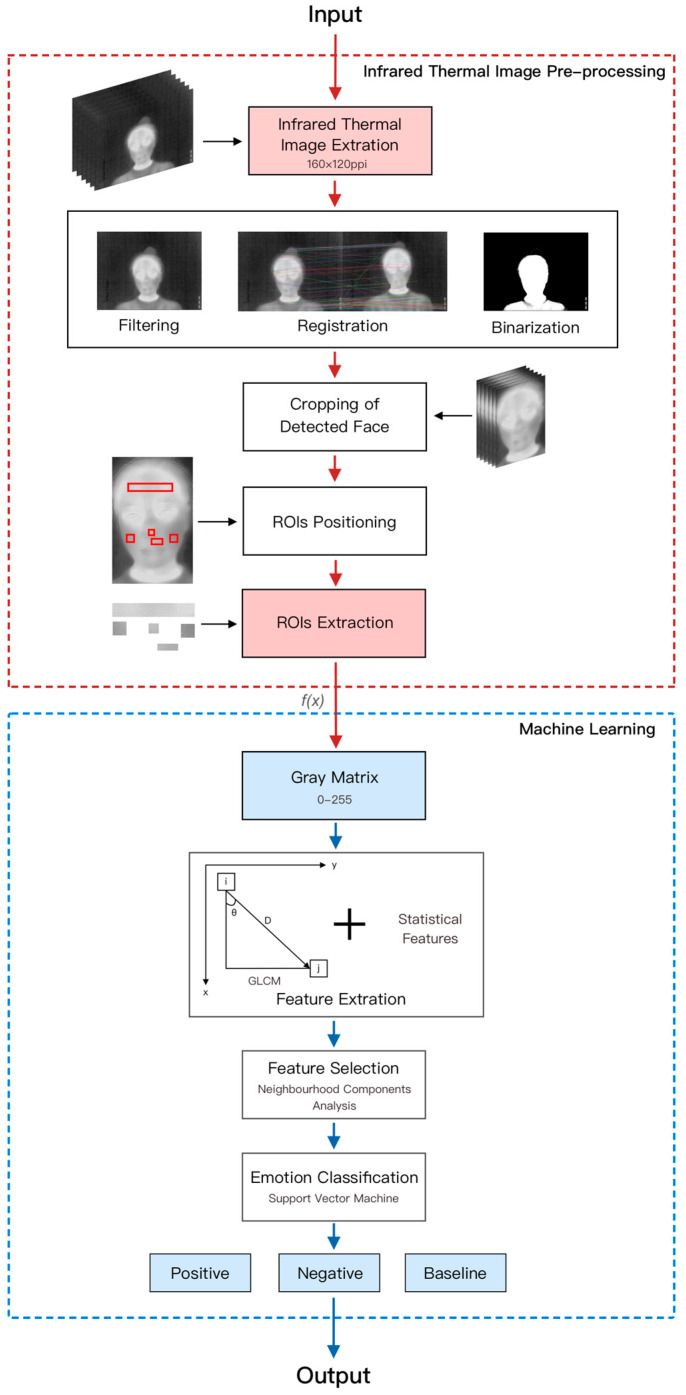
Thermal experiment data processing.

To avoid any interference from the use of a fixed head device, such a device was not used in this experiment. Therefore, registration was applied to eliminate the deviation caused by head movement. The centroid of the eye area was positioned in fixed images used for registration [41] since only the position of the image was translated or rotated, and the gray matrix was not changed. Subsequently, median and Gaussian filters were used to eliminate the noise in the registration image to obtain the best-binarized images, i.e., images that display the facial contour of the participant. Then, a box was used to frame the face, and the original image was generated in batches for further statistical calculation. The forehead, left cheek, right cheek, nose, and maxillary are the 5 regions of interest (ROIs) that are frequently used in emotion research using IRTIs and have yielded significant results [42,43,44]. The ROIs were located with a geometric model of the face, the face width was represented by D, and the regions to be studied were obtained according to the center of the geometric ratio of the ROIs [28,45]. The first frame of each group of thermal images was positioned manually. Afterward, the ROI selection box automatically located the ROIs of each frame. Finally, all ROIs of each frame were accurately located. Figure 5 describes the entire process of thermal data processing.

#### 2.6.2. Feature Extraction

The IRTIs data of participants at baseline and when using the different websites were extracted. Afterward, MATLAB R2020b (MathWorks, Natick, MA, USA) was used to convert each thermal image into a gray matrix. Then, the statistical features and the texture features of the GLCM were calculated, as described below.

The statistical features were obtained from extracted thermal imaging data features in the original gray matrix. In the following equations, $M^{k}\in M^{w\times h}$ is an ROI described by a series of pixels $M_{ij}$ in the range of 0–255 (grayscale 8 bits), *k* is the currently processed ROI, and *K* is the number of ROIs.

(1) $$f_{1}=\;\bar{M}\;=\frac{1}{w\cdot h}\sum_{i=1}^{w}\sum_{j=1}^{h}M_{ij},$$

where $f_{1}$ is the mean values of all pixels $M_{ij}$, and *w* and *h* represent the rows and columns of the gray matrix, respectively.

(2) $$f_{2}=\sigma^{2}=\frac{1}{\left(w\cdot h\right)-1}\sum_{i=1}^{w}\sum_{j=1}^{h}{\left(M_{ij}-\bar{M}\right)}^{2},$$

where $f_{2}$ shows the variance in all pixels.

(3) $$f_{3}=\frac{1}{w}\sum_{i=1}^{w}\frac{1}{\left(h-1\right)}\sum_{j=1}^{h}{\left(M_{ij}-{\bar{M}}_{i}\right)}^{2},$$

where $f_{3}$ represents the mean value of the variance in each row in the current gray matrix and ${\bar{M}}_{i}$ is the average value of row *i*.

(4) $$f_{4}=\frac{1}{h}\sum_{j=1}^{h}\frac{1}{\left(w-1\right)}\sum_{i=1}^{w}{\left(M_{ij}-{\bar{M}}_{j}\right)}^{2},$$

where $f_{4\;}$ represents the mean value of the variance in each column in the current gray matrix and ${\bar{M}}_{j}$ is the average value of column *j*.

In addition, $f_{5}$ represents the contrast of all pixels $M_{ij}$, $f_{6}$ is the median value of all gray matrices, and $f_{7}$ and $f_{8}$ represent the median values of each row and column, respectively.

(5) $$f_{5}=max\left(M\right)-min\left(M\right),$$

(6) $$f_{6}=median\left(M\right),$$

(7) $$f_{7}=\frac{1}{w}\sum_{i=1}^{w}median\left(M_{i}\right),$$

(8) $$f_{8}=\frac{1}{h}\sum_{j=1}^{h}median\left(M_{j}\right),$$

The GLCM is an extensive image texture analysis method that relies on angle and distance. Five texture feature statistics of the GLCM were used, as described below.

Let an image have $N_{x}$ columns and $N_{y}$ rows. The gray level that occurs in each pixel is quantized as $N_{g}$ levels. Let $L_{x}=\left\{1,2,\ldots ,N_{x}\right\}$ be the columns and $L_{y}=\left\{1,2,\ldots ,N_{y}\right\}$ be the rows; then, $L_{x}\times L_{y}\;$is a set of pixels. Image *I* is a function that assigns some gray levels in $G=\left\{1,2,\ldots ,N_{g}\right\}$ to every pixel; $I:L_{x}\times L_{y}\to G$. The texture-context information is specified by the matrix of relative frequencies $P_{ij}$, with two neighboring pixels separated by distance *d* occurring on the image, one with gray level i and the other with gray level j. Such matrices of gray-level co-occurrence frequencies are a function of the angular relationship and distance between the neighboring pixels. Formally, for angles quantized to 45° intervals, the unnormalized frequencies are defined by

$$P(i,j,d,0{}^{\circ})=\#\{(\left(a,b\right),\left(e,f\right))\in (L_{x}\times L_{y})\times (L_{x}\times L_{y})|a-e=0,\;|b-f|=d,\;I(a,b)=i,\;I(e,f)=j\},$$

$$\begin{array}{cc} P(i,j,d,45{}^{\circ})= & \#\{(\left(a,b\right),\left(e,f\right))\in (L_{x}\times L_{y})\times (L_{x}\times L_{y})|(a-e=d,\;b-f=-d)\;or\;(a-e=-d,\;b-f\\ & =d),\;I(a,b)=i,\;I(e,f)=j\} \end{array}$$

$$P(i,j,d,90{}^{\circ})=\#\{(\left(a,b\right),\left(e,f\right))\in (L_{x}\times L_{y})\times (L_{x}\times L_{y})||a-e|=d,\;b-f=0,\;I(a,b)=i,\;I(e,f)=j\}$$

(9) $$\begin{array}{cc} P(i,j,d,135{}^{\circ})= & \#\{(\left(a,b\right),\left(e,f\right))\in (L_{x}\times L_{y})\times (L_{x}\times L_{y})|(a-e=d,\;b-f=d)\;or\;(a-e\\ \; & =-d,\;b-f=-d),\;I(a,b)=i,\;I(e,f)=j\} \end{array}$$

where # denotes the number of elements in the set.

Let p(i,j) be the (i,j)th entry in the GLCM. The means and standard deviations for the columns and rows of the matrix are

(10) $$\mu_{x}=\;\sum_{i=1}^{N_{g}}\sum_{j=1}^{N_{g}}i\cdot p(i,j),$$

(11) $$\mu_{y}=\;\sum_{i=1}^{N_{g}}\sum_{j=1}^{N_{g}}j\cdot p(i,j),$$

(12) $$\sigma_{x}=\sum_{i=1}^{N_{g}}\sum_{j=1}^{N_{g}}{\left(i-\mu_{x}\right)}^{2}\cdot p\left(i,j\right),$$

(13) $$\sigma_{y}=\sum_{i=1}^{N_{g}}\sum_{j=1}^{N_{g}}{\left(j-\mu_{y}\right)}^{2}\cdot p\left(i,j\right)$$

(14) $$f_{9}=\sum_{i=1}^{N_{g}}\sum_{j=1}^{N_{g}}p{\left(i,j\right)}^{2},$$

(15) $$f_{10}=\sum_{n=0}^{N_{g}-1}n^{2}\left\{\sum_{i=1}^{N_{g}}\sum_{j=1}^{N_{g}}p\left(i,j\right)|\left|i-j\right|=n\right\},$$

(16) $$f_{11}=\frac{\sum_{i=1}^{N_{g}}\sum_{j=1}^{N_{g}}\left(ij\right)p\left(i,j\right)-\mu_{x}\mu_{y}}{\sigma_{x}\sigma_{y}},$$

(17) $$f_{12}=\sum_{i=1}^{N_{g}}\sum_{j=1}^{N_{g}}\frac{1}{1+{\left(i-j\right)}^{2}}\;p\left(i,j\right),$$

(18) $$f_{13}=\sum_{i=1}^{N_{g}}\sum_{j=1}^{N_{g}}\left|i-j\right|\cdot \;p\left(i,j\right),$$

(19) $$f_{c+5}=f_{c}\left(k\right)\left|\right.d=2,4,8,16,\;9\leq k\leq K,\;c\geq 9$$

where $f_{9},f_{10},f_{11},f_{12},\;and\;f_{13}$ represent the angular second moment (ASM), contrast, correlation, homogeneity, and dissimilarity of the GLCM features, respectively [46]. Finally, these 5 features change the angle and distance (in our study d = 2, 4, 8, and 16). Therefore, 16 features are derived from each of the above features, as shown in Equation (19), where *k* is the current ROI being processed.

In summary, each ROI has 8 statistical features and 80 GLCM texture features. Therefore, each region has a total of 88 features, and the total number of features is 440.

#### 2.6.3. Feature Selection

Relevant features that facilitated classification needed to be selected from all features to avoid the dimensionality problem. Hence, the neighborhood component analysis (NCA) method was used [47,48], and the feature weight was used to maximize the expected classification accuracy through regularization. In this process, fivefold cross-validation was used to tune the parameters of λ in the regularization item, and the minimum loss value was determined according to the loss function.

Let $T=\left\{\left(x_{1},y_{1},\right),\ldots ,\left(x_{i},y_{i},\right),\ldots ,\left(x_{N},y_{N}\right)\right\}$ be the training set, where $x_{i}\;$is a *d*-dimensional feature vector, $y_{i}\in \left\{1,2,...,C\right\}$ is the corresponding training target, and N represents numerous samples. To select the optimal feature, we need a weight vector *w*, and the weighted Mahalanobis distance between two samples $x_{i}$ and $x_{j}$ is:

(20) $$D_{w}\left(x_{i},y_{i},\right)=\sum_{l=1}^{d}\omega_{l}^{2}\left|x_{il}-x_{jl}\right|,$$

where $\omega_{l}$ is a weight associated with the *l*th feature. Since it is a nondifferentiable function to confirm the nearest neighbor as the reference point with the leave-one-out method, an approximate probability distribution was used to determine the reference point. Therefore, the probability that $x_{i}$ chooses $x_{j}$ as the reference point is:

(21) $$p_{ij}=\left\{\begin{array}{c} \frac{k\left(D_{w}\left(x_{i,}x_{j}\right)\right)}{\sum_{k\neq i}k\left(D_{w}\left(x_{i,}x_{k}\right)\right)},if\;i\neq j\\ 0,\;if\;i=j \end{array}\right.$$

where k(z)=exp(−z/σ) is a kernel function and σ is the kernel width. If σ→0, only the nearest neighbor of the query sample is selected as its reference point; however, if σ→+∞, all of the points have the same chance of being selected apart from the query point. Thus, Equation (22) shows the probability that query point $x_{i}$ is correctly classified.

(22) $$p_{i}=\sum_{j}y_{ij}p_{ij}$$

Afterward, to avoid overfitting and introduce a regularization term λ, the classification accuracy of the leave-one-out method is obtained as:

(23) $$\xi (w)=\sum_{i}\sum_{j}y_{ij}p_{ij}-\lambda \sum_{l=1}^{d}\omega_{l}^{2}$$

Finally, the function ξ(w) is differentiable, and its derivative with respect to $\omega_{l}\;$can be computed:

(24) $$\frac{\partial \xi (w)}{\partial \omega_{l}}=2(\frac{1}{\sigma }\sum_{i}(p_{i}\sum_{j\neq i}p_{ij}\left|x_{il}-x_{jl}\right|-\sum_{j}y_{ij}p_{ij}\left|x_{il}-x_{jl}\right|)-\lambda )\omega_{l}$$

#### 2.6.4. Emotional Classification

To eliminate the influence of the data unit, the feature was normalized by the *z* score, and the formula is shown in Equation (25)

(25) $$x^{*}=\frac{x-\mu }{\sigma },$$

where *x* is the original data, $x^{*}$ is the normalized data, and μ and σ are the mean and standard deviation of the original data *x*, respectively.

The labels of the data used for machine learning were calibrated according to the valence reported by participants on the SAM. We only classified two emotional experiences, positive and negative. Therefore, when the valence was less than 3, the emotional experience was classified as a negative; otherwise, it was classified as positive. Baseline served as an effective point of comparison for emotional experiences. Consequently, three binary classification tasks were identified: positive emotional experiences versus baseline (P-Base), negative emotional experiences versus baseline (N-Base), and positive emotional experiences versus negative emotional experiences (P-N). Based on the results of feature selection, we selected the top 15 features with the highest selection frequency in each task.

The SVM model, also known as a supervised learning approach, was chosen to classify the emotional experiences. The application of this classifier to binary classification tasks in machine learning is more mature. The SVM model identifies a high-dimensional discriminative hyperplane that can distinguish two categories of the datasets and maximize their differences. The performance of the SVM model can be further improved by optimizing parameters and selecting different kernel functions. In this experiment, the cross-validation method and grid search were used to train the SVM model to determine the optimal parameters and kernel function. In addition, we found that the SVM model performed best with a Gaussian kernel function and a parameter of 2.15 in this research.

### 2.7. Statistical Analysis

The variation in the valence and arousal dimensions from the SAM were evaluated according to the means and standard deviations; therefore, the emotional experiences were classified according to the experimental results of the subjective evaluation. The mean grayscale value variations in each ROI between the baseline and the period of the tasks were compared. Student’s *t*-tests (α = 0.05) with Bonferroni correction were used to verify the significance of emotional experience fluctuation. To mitigate the impact of uneven data distribution, this study employed the F1−score, in addition to the accuracy, as an evaluation metric for assessing the classification of emotional experiences. The F1−score is the weighted average of precision and recall in machine learning. The F1−score is denoted by the following, Equation (28), where TP is the number of true positives, FP is the number of false positives, and FN is the number of false negatives [49].

(26) $$precision=\frac{TP}{TP+FP}\;,$$

(27) $$recall=\frac{TP}{TP+FN}\;,$$

(28) $$F1-score=2\times \frac{precision\times recall}{precision+recall}\;.$$

## 3. Results

### 3.1. SAM Data

The valence and arousal of the 24 participants were recorded after completing each task. To check whether the emotional experiences were successfully manipulated, we performed a two-way analysis of variance (ANOVA), with valence and arousal as the dependent variables. The consequence was that the differences in valence for different usability and aesthetic elements were significant (usability: *F* (1,22) = 122.525, *p* < 0.001, and $\eta^{2}$ = 0.571; aesthetics: *F* (1,22) = 10.423, *p* = 0.002, and $\eta^{2}$ = 0.102), and the differences in arousal were not significant (usability: *F* (1,22) = 0.951, *p* = 0.332, and $\eta^{2}$ = 0.010; aesthetics: *F* (1,22) = 0.385, *p* = 0.536, and $\eta^{2}$ = 0.004). In addition, no interaction effects between the usability and aesthetics on either the valence or arousal were found (valence: *p* = 0.49, arousal: *p* = 0.66).

The calculated means and standard deviations are shown in Table 2. Positive emotional experiences were obtained when using both U+A+ and U+A− websites with a valence greater than or equal to 3, while negative emotional experiences were obtained when using U−A+ and U−A− websites with a valence less than 3. However, an arousal score of approximately 3 was obtained when using all websites. The emotional changes during each interaction were not as strong as those from the video–picture stimuli. Thus, the arousal level did not fluctuate much. Although the arousal score variation for the four websites was not substantial, the average arousal rating exceeded 3, indicating a high arousal. This indicates that the participants experienced emotional responses.

The SAM valence scores were considered the ground truth classification of the emotional experiences. Based on the valence results, the participants’ emotional experiences were categorized into two categories: positive and negative. The participants had positive emotional experiences when using the U+A+ and U+A− websites and negative emotional experiences when using the U−A+ and U−A− websites.

### 3.2. Thermal Data

#### 3.2.1. Feature Selection

**Figure 6:**
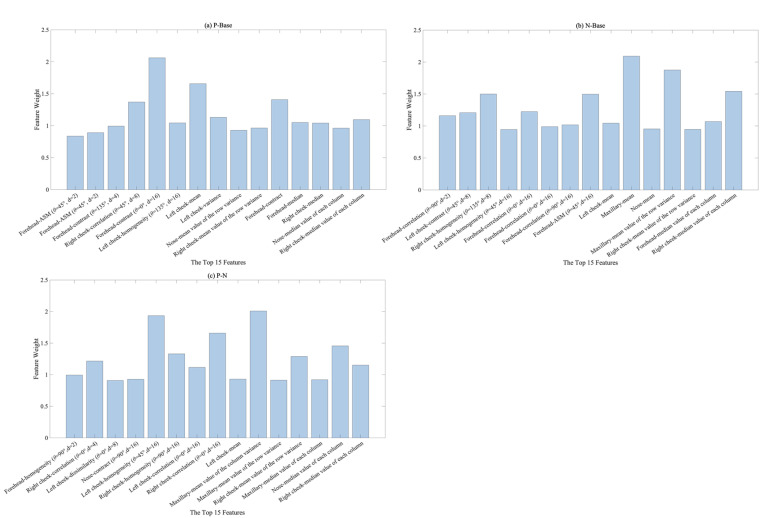
Results of feature selection based on NCA.

Data from one participant with excessive head movements were excluded, and the dataset containing data from the remaining 23 participants was used for machine learning. NCA was used for feature selection, and the weights of each feature for the three binary classification tasks (positive emotional experiences versus baseline, negative emotional experiences versus baseline, and positive emotional experiences versus negative emotional experiences) were calculated. Subsequently, based on the weights of the 440 features in each binary classification task, the top 15 features with the highest weights were selected for subsequent model training. The highest weight among the selected features was 2.09, and the lowest was 0.84, as shown in Figure 6.

**Figure 7:**
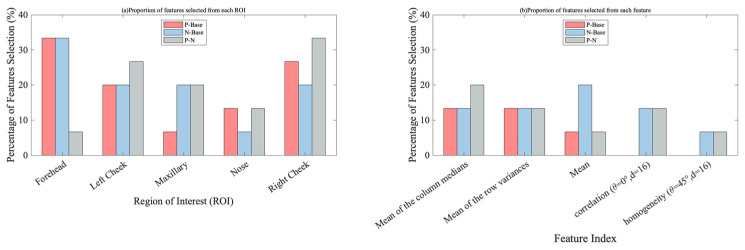
Proportion of features selected from each ROI and feature.

Table 3 summarizes the feature selection results for the three binary classification tasks. Figure 7a shows the proportion of each ROI in terms of feature selection for the different classification tasks. The features selected for classification were mostly concentrated on the forehead, left cheek, and right cheek. The highest proportion of features was selected around the right cheek. Simultaneously, Figure 7b describes the five features with the highest percentages of selection: mean of the column medians, mean of the row variances, mean, correlation (θ=0°, d=16), and homogeneity (θ=45°, d=16). According to Table 3, there were three features selected in all three binary classification tasks, namely left cheek-mean, right cheek-mean value of the row variance, and right cheek-median value of each column.

#### 3.2.2. Emotional Experiences Classification

The data allocation ratio for the training and test sets was 5:1, and fivefold cross-validation was performed with the training set to effectively select the classifier that performed best with the dataset as well as to prevent overfitting. As shown in Table 4, the dataset was divided into four groups according to the order in which the experiments were performed, and the data of the last participant in each group were classified as the test set. The SVM classifier was used to train and test these data based on 15 features. To avoid the effects of uneven data distribution, the F1−score was calculated in this study in addition to the accuracy of the classification, as shown in Table 5.

In the three binary classification tasks of P-Base, N-Base, and P-N, the SVM classifier was more effective in classifying different emotional experiences and baselines, with a mean accuracy and F1−score above 0.75. However, the F1−score for the classification of positive and negative emotional experiences was lower.

#### 3.2.3. Facial Grayscale Data Variation

**Figure 8:**
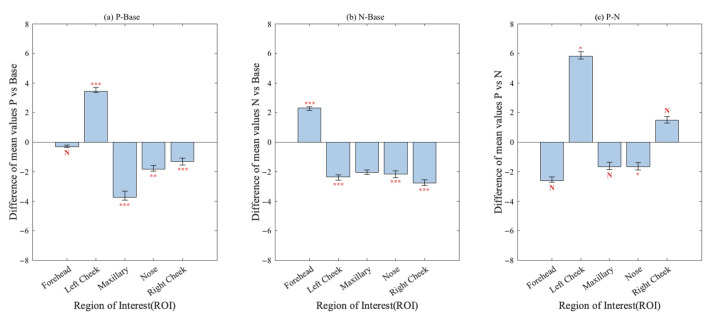
The mean grayscale value difference of the five ROIs under the negative emotional experience, positive emotional experience, and baseline, and the significant analysis results of Student’s test. P vs. Base means positive emotional experience versus baseline, N vs. Base means negative emotional experience versus baseline, P vs. N means positive emotional experience versus negative emotional experience. * (*p* ≤ 0.05), ** (*p* ≤ 0.01), and *** (*p* ≤ 0.001) indicate significance, and N means not significant.

Figure 8 illustrates variations in the mean grayscale value across five ROIs among participants in three emotional states: positive emotional experience, negative emotional experience, and baseline. These thermal trends encompass increments (positive value), decrements (negative value), and stability. Significance testing for these changes was verified according to Student’s *t*-test.

As a result, we observed significant alterations in the grayscale value within the maxillary, nose, and right cheek when comparing positive and negative emotional experiences to the baseline. Furthermore, the grayscale value of the left cheek increased during positive emotional experiences, while the value decreased during negative emotional experiences, and all of the changes were significant. The grayscale value of the forehead increased significantly during negative emotional experiences, and the grayscale value was relatively stable when experiencing positive emotional experiences. Comparing the differences between positive and negative emotional experiences, the grayscale value of the left cheek increased significantly, and the grayscale value of the nose decreased significantly, while no statistically significant changes were observed in other regions.

## 4. Discussion

### 4.1. Valence and Arousal Analysis of Emotional Experiences

By requiring participants to perform tasks on different websites, our objective was to elicit emotional experiences within the realm of HCI. Prior research has established that website usability and aesthetics can trigger user emotions [50,51,52]. Performing tasks on a website with design flaws can lead to negative emotional experiences for users, while executing tasks on a perfectly designed website can result in a positive emotional experience. It has also been confirmed that emotional experience changes induced by usability also impact users’ degrees of physiological activation. The results from the SAM showed that manipulating the usability and aesthetics of B2C websites altered the valence and arousal of emotional experiences. As depicted in Table 2, websites with high usability and high aesthetics seemed to provide the most pleasant experiences for users, and websites with low usability and low aesthetics seemed to provide the most unpleasant experiences for users. The change in valence was significant, while the change in arousal was not. Valence is an effective dimension for classifying user emotional experience. This finding is consistent with previous emotion research in which eliciting arousal was more difficult than eliciting a specific valence [34,53]. This may be because the websites that we designed may not have had the necessary elements to elicit differences in arousal, and the experiment may have lacked conditions that induced arousal. In contrast, requiring participants to use poorly designed and unfamiliar websites created a level of frustration that affected the significance of the differences in valence [54]. This result influenced the subsequent classification of emotional experiences.

### 4.2. Feature Selection and Classification

As shown in Figure 7a, the NCA feature selection results revealed that the forehead, left check, and right cheek features were selected the most times, and the right cheek was the ROI with the highest contribution, accounting for 26% of the total features. The left cheek mean and right cheek mean values of the row variance, and right cheek median value of each column, were selected in all three binary classification tasks, which also showed that the right and left cheek were important ROIs in the face. Previous research on the thermography of human faces has also achieved related results [31,45,55]. This shows that these three ROIs are more closely related to changes in emotional experiences. Thus, they are the key ROIs for item feature selection. The mean of the column medians, mean of the row variances, and mean were the statistical features that were most frequently selected, demonstrating that the extraction of statistical features from IRTI data is important for the classification of emotional experiences. The correlation (θ=0°,d=16) and homogeneity (θ=45°,d=16) GLCM texture features were also selected with a high frequency, as shown in Figure 7b. This illustrates the effectiveness of GLCM texture feature extraction, which can provide effective feature information for the classification of emotional experiences based on IRTIs [18,56]. Furthermore, these research results illustrated that the forehead, left cheek, and right cheek are the ROIs to prioritize for feature extraction and that GLCM texture features could be effectively applied in research on emotional experience classification based on IRTIs.

There have been many studies using SVM models to classify emotions in thermal images [57,58]. An SVM model was chosen for the classification of emotional experiences, and this research was based on cross-subject training. As shown in Table 4 and Table 5, the mean classification accuracy and F1−score were 0.7807 and 0.7786 for positive emotional experiences and the baseline, respectively, 0.7697 and 0.7640 for negative emotional experiences and the baseline, and 0.5472 and 0.4694 for positive emotional experiences and negative emotional experiences, respectively. Thus, it was concluded that the SVM classifier was effective in classifying emotional experiences and the baseline; however, it was unable to effectively classify positive and negative emotional experiences. According to the theory of emotional dimensions, there exists the arousal effect from baseline to emotional experiences, as well as the valence effect from positive emotional experiences to negative emotional experiences. Consistent with previous experimental findings, we also observed an arousal effect without a valence effect. This suggested that participants’ responses at baseline differ from those in emotional experiences, and that there is no distinction in responses between positive and negative emotions [59]. Furthermore, it appeared that arousal may be a significant dimension for eliciting physiological changes in users due to emotional stimuli [60]. The performance of the SVM classifier was attributed to the underlying physiology and the associated experimental protocol. The degree of activation of facial thermosensitivity is very different in the baseline and emotional experiences. Thermal print due to emotional stimuli is mainly manifested by subcutaneous vasoconstriction and emotional sweating. In accordance with this point, better classification accuracy was obtained for the classification of the baseline and emotional experiences. At the same time, the strength of the emotional stimuli appeared to affect the intensity of the manifestation of thermal changes in the skin, and since the stimuli in the experiment did not cause sufficient intensity differences in valence and arousal, this may have resulted in the inability to measure them with IRTIs [61]. To effectively classify positive and negative emotional experiences in future experimental designs, it is imperative to augment the intensity and contrast of experimental stimuli and improve emotional classification algorithms. Nonetheless, this experiment reveals the effectiveness of baseline- and emotion-level classification.

Since the classification tasks were cross-subject, they were validated based on multiple participants, and all accuracy rates in both the baseline and emotional experience classification tasks exceed 70%. This suggests that the selected features may be the most robust among all features, representing the most natural responses of participants to stimuli. However, for future validation of the classification experiments, it is essential to expand the dataset.

### 4.3. ROI Trends for Different Emotional Experiences

Figure 8 shows the change trend and significance of each ROI for negative and positive emotional experiences. There was a significant increase in grayscale data in the forehead during negative emotional experiences. This may be due to the negative emotion of stress that the participants experienced while using the poor websites to complete tasks, which led to increases in blood flow to the forehead and concentration in the vascular system of the forehead, thus increasing the grayscale data [62]. A decrease in cheek grayscale data is a marker of negative emotion [63]. The same thermal trend was found in this study. This condition is thought to be caused by partial adrenergic blood flow to a more important facial region and the result of emotional sweating [28]. The nose is considered the most reliable facial region for identifying emotions. There was a significant decrease in the grayscale data of the nose under both negative and positive emotional experiences. This occurrence was attributed to the activation of the sympathetic nervous system, thus restricting blood flow to the vascular surface, which is also known as the vasoconstriction mechanism. In contrast, the decrease in grayscale data in the maxillary region was mainly due to the activation of the sweat glands. Changes in these ROIs can better inform analysis of the thermal trends in different emotional experiences and improve classification.

## 5. Conclusions

In this paper, IRTIs were used in HCI research. In contrast to previous emotional stimulation research, in this study, B2C websites were used as experimental materials to explore the emotional changes in participants during a task. The results demonstrated that thermal imaging data can effectively reflect the changes in the emotional experience of users interacting with websites with different designs. The main conclusions of this study are as follows. We found that, when participants used different websites, they exhibited greater changes in valence than in arousal. Therefore, we used valence as a benchmark to divide user emotional experiences into positive or negative experiences. In the feature selection process, the left cheek, right cheek, and forehead were the three ROIs that contributed the most features, while the mean of the column medians, the mean of the row variances, the mean in the statistical features, and the correlation and homogeneity in the GLCM texture feature were the most-selected features. In the feature classification process, we found that the SVM model demonstrates good classification performance between baseline and emotional experiences. The results of this study proved the effectiveness of applying IRTIs in HCI research and illuminate more research directions for the application of IRTIs.

There are also some limitations of this study. First, regarding the design of the experimental stimuli, only three design elements, IAs, website background color, and product display shape, were manipulated in this study. There are many other design elements that affect users’ emotional experiences on websites. In future research, additional design elements can be manipulated to elicit users’ emotions. Second, in this experiment, only the changes in emotion of five ROIs were verified, and increasing the number of ROIs to better measure and classify emotions should be considered. Third, the ROI extraction was semiautomated. Due to the inevitable head movements of participants during the experiment, real-time tracking and positioning of the facial ROIs could enhance the accuracy of data extraction.

## Figures and Tables

**Table 1:** Latent semantic analysis: means and standard deviations (SD) of information scent for navigation paths to target or non-target.

	Paths to Target		Paths to Non-Target	
	Mean	SD	Mean	SD
Good IAs	0.42	0.09	0.21	0.07
Poor IAs	0.19	0.09	0.18	0.06

**Table 2:** Means (M) and standard deviations (SD) of the SAM.

	Valence		Arousal	
	M	SD	M	SD
U+A+	4.74	0.62	3.41	1.00
U+A−	4.04	0.89	3.68	1.07
U−A+	2.52	1.04	3.22	1.01
U−A−	2.09	0.86	3.81	1.38

**Table 3:** Features with the highest weights for classification of emotional experiences.

Top 15 Features for P-Base	Top 15 Features for N-Base	Top 15 Features for P-N
■ Maxillary-correlation (θ=0°, d=2)	■ Forehead-correlation (θ=90°, d=2)	■ Forehead-homogeneity (θ=90°, d=2)
■ Forehead-ASM (θ=45°, d=2)	■ Left cheek-contrast (θ=45°, d=8)	■ Right cheek-correlation (θ=0°, d=4)
■ Forehead-contrast (θ=135°, d=4)	■ Right cheek-homogeneity (θ=135°, d=8)	■ Left cheek-dissimilarity (θ=0°, d=8)
■ Right cheek-correlation (θ=45°, d=8)	■ Left cheek-homogeneity (θ=45°, d=16)	■ Nose-contrast (θ=90°, d=16)
■ Forehead-contrast (θ=0°, d=16)	■ Forehead-correlation (θ=0°, d=16)	■ Left cheek-homogeneity (θ=45°, d=16)
■ Left cheek-homogeneity (θ=135°, d=16)	■ Maxillary-correlation (θ=0°, d=16)	■ Right cheek-homogeneity (θ=90°, d=16)
■ Left cheek-mean	■ Forehead-correlation (θ=90°, d=16)	■ Left cheek-correlation (θ=0°, d=16)
■ Left cheek-variance	■ Forehead-ASM (θ=45°, d=16)	■ Right cheek-correlation (θ=0°, d=16)
■ Nose-mean value of the row variance	■ Left cheek-mean	■ Left cheek-mean
■ Right cheek-mean value of the row variance	■ Maxillary-mean	■ Maxillary-mean value of the column variance
■ Forehead-contrast	■ Nose-mean	■ Maxillary-mean value of the row variance
■ Forehead-median	■ Maxillary-mean value of the row variance	■ Right cheek-mean value of the row variance
■ Right cheek-median	■ Right cheek-mean value of the row variance	■ Maxillary-median value of each column
■ Nose-median value of each column	■ Forehead-median value of each column	■ Nose-median value of each column
■ Right cheek-median value of each column	■ Right cheek-median value of each column	■ Right cheek-median value of each column

**Table 4:** Classification accuracy for the three binary classification tasks.

	Emotional Experiences		
	P-Base	N-Base	P-N
S1, S2, S3, S4, S5 and S7	0.7778	0.7500	0.5000
S8, S9, S10, S11, S12 and S13	0.7895	0.7500	0.6316
S14, S15, S16, S17, S18 and S19	0.8333	0.8621	0.5385
S20, S21, S22, S23 and S24	0.7222	0.7167	0.5185
Mean accuracy	0.7807	0.7697	0.5472

**Table 5:** F1−score for three binary classification tasks.

	Emotional Experiences		
	P-Base	N-Base	P-N
S1, S2, S3, S4, S5 and S7	0.7784	0.7499	0.4905
S8, S9, S10, S11, S12 and S13	0.7896	0.7333	0.6311
S14, S15, S16, S17, S18 and S19	0.8329	0.8601	0.3769
S20, S21, S22, S23 and S24	0.7134	0.7128	0.3794
Mean F1−score	0.7786	0.7640	0.4694

## Data Availability

The data are not publicly available due to privacy restrictions.

## Abbreviations

IRTIs	infrared thermal images
B2C	business-to-consumer
SVM	support vector machine
ROIs	regions of interest
HCI	human-computer interaction
SAM	Self-Assessment Manikin
SCL	skin conductance level
FT	fingertip temperature
HR	heart rate
BVP	blood volume pulse
EDA	electrodermal activity
GSR	galvanic skin response
SKT	skin temperature
RSP	respiration rate
ERPs	event-related potentials
EEG	electroencephalogram
ECG	electrocardiograms
ANS	autonomic nervous system
SVM	support vector machine
GLCM	gray-level cooccurrence matrix
IAs	information architectures
LSA	latent semantic analysis
M	means
SD	standard deviations
U+A+	high usability and high aesthetics
U+A−	high usability and low aesthetics
U−A+	low usability and high aesthetics
U−A−	low usability and low aesthetics
ASM	angular second moment
NCA	neighborhood component analysis
TP	true positive
FP	false positive
FN	false negative
ANOVA	analysis of variance
P-Base	positive emotional experiences versus baseline
N-Base	negative emotional experiences versus baseline
P-N	negative emotional experiences versus baseline
